# Introduction to the 7^th ^Annual Conference on the Science of Dissemination and Implementation: transforming health systems to optimize individual and population health

**DOI:** 10.1186/1748-5908-10-S1-I1

**Published:** 2015-08-20

**Authors:** David Chambers, Lisa Simpson

**Affiliations:** 1 Division of Cancer Control and Population Sciences, National Cancer Institute, National Institutes of Health, Rockville, MD, 20850, USA; 2 AcademyHealth, Washington, D.C., 20036, USA

## 

While scientists and practitioners have long been interested in the best ways to use scientific data to improve health care, the past decade has seen significant growth in interest and activity in dissemination and implementation research in health, as evidenced by expansion of conceptual and empirical work in the area. Funding opportunities at the National Institutes of Health (NIH) have defined dissemination as "the targeted distribution of information and intervention materials to a specific public health or clinical practice audience," and implementation as "the use of strategies to adopt and integrate evidence-based health interventions and change practice patterns within specific settings," (http://grants.nih.gov/grants/guide/pa-files/PAR-13-055.html). Research on these key processes is predicated on establishing the appropriate fit between scientific findings, research-tested interventions, and the contexts in which health and health care are optimized.

One reflection of this growth has been the significant participation of the research community in a series of annual meetings on the science of dissemination and implementation. These meetings, initially organized by the NIH along with the US Department of Veterans Affairs (VA), were established as a way for the scientific community to come together, explore challenges and successes in the field, and offer opportunities for technical assistance and training for newcomers to the field. After five annual meetings, conference planners recognized that the interest in dissemination and implementation research extended far beyond the two organizations responsible, and budgetary constraints would require a new model for the conference that would leverage activities and interest in the growing field, while building a model for sustainability that could put the meeting series on firm ground for the years to come.

A new partnership was formed between the National Institutes of Health and AcademyHealth to organize and co-host the 7th Annual Conference on the Science of Dissemination and Implementation (http://www.academyhealth.org/Events/events.cfm?ItemNumber=14130&navItemNumber=14309). NIH reconnected with past sponsors-Agency for Healthcare Research and Quality (AHRQ) and the VA, while AcademyHealth enlisted additional Non-Federal partners-the Patient Centered Outcomes Research Institute (PCORI), the Robert Wood Johnson Foundation (RWJF), and the WT Grant Foundation. NIH and AcademyHealth co-led the program planning committee, an executive committee of principals from the partnering organizations, and convened a scientific advisory panel to suggest plenary speakers and advise on the call for abstracts.

The meeting was highlighted by a keynote talk from Peter Pronovost, M.D., Ph.D, Johns Hopkins University, who described the pathway from observation about the prevalence of hospital-acquired infections, through the development of interventions to dramatically reduce them, to the implementation of those interventions to improve safety in hospital systems around the world. The meeting also featured three plenary sessions, one discussing the bridge between dissemination and implementation research and health disparities, a second focusing on next steps for the research community, and a final one soliciting stakeholder involvement to improve the relevance of research studies to health systems, communities, and other stakeholders. In addition, the meeting included a number of lunchtime discussion forums, a technical assistance workshop for investigators interested in submitting grants to NIH, AHRQ, Centers for Disease Control and Prevention (CDC), PCORI, RWJF, and the VA.

The call for abstracts, which solicited submissions for individual paper presentations, individual posters, and panel presentations, resulted in 660 submissions, almost twice as many as were received in previous years. Over one hundred scientific reviewers from the program planning committee and scientific community provided the expertise to assess the quality of the abstracts. For the final program, 79 oral presentations, 12 panels, and 60 posters were accepted. An additional opportunity to present was afforded to submitters with the development of a "virtual presentation library," where PDFs of slide presentations were made available at the meeting on local computers.

The abstracts accepted for oral presentations reflect the breadth and depth of the field of dissemination and implementation science today. The settings in which D & I science is being conducted include the full range of settings where children and adults receive care: primary care, safety net provider systems, long term care, community mental health, occupational health, correctional facilities and juvenile justice, home based care, churches, and early care and education. A number of these examined care for underserved populations. Somewhat underrepresented were studies of public and population health interventions, and studies at the broader community level.

Wide variation in the scale of the interventions tested was found: from a study with only nine practices to one working to change behaviors for 8,000 staff. Similarly, the range of intervention effect sizes also varied greatly from many with only modest effects (less than 10 percent change) to a few with improvements nearing 60 percent (e.g. improved viral suppression in patients with HIV). Several abstracts leveraged technology to enhance implementation, including texting, tablet based risk assessment to tailor interventions and maximize uptake, and reminder systems. Others used more resource intensive approaches such as academic detailing, nurse case managers, and community health workers. A number of abstracts added to our methods, frameworks and tools by validating existing tools, testing existing frameworks in different contexts or developing new ones. Throughout, the abstracts revealed how diverse and multidisciplinary this field is and the importance of mixed methods (e.g. the role of deep ethnographic work to understand how implementation happens).

Two critical aspects of implementation science that were not well represented in the presentations were evaluations of the costs of implementation or of efforts to de-implement. In most settings, including health care settings, resources are constrained and decision-makers must make choices about which practices to implement and which to stop doing. The field has an opportunity to help inform these decisions as well. We hope to see more on these topics in the next annual meeting in 2015.

A final dimension of the breadth and depth of the field is the variety of research funders represented. While a sizable number of the studies presented were funded by the NIH, numerous studies had funding from other governmental and private sources, including the VA, AHRQ, the Centers for Medicare and Medicaid Services, the US Agency for International Development, the UK National Institute for Health Research, private foundations, voluntary health associations, as well as state health departments.

The final meeting agenda, with many of the slides used in presentations, is available at: http://www.academyhealth.org/Events/events.cfm?ItemNumber=14130&navItemNumber=14309

This supplement has compiled the abstracts for presented papers and panel sessions from the 7th Annual Meeting on the Science of Dissemination and Implementation in Health: Transforming Health Systems to Optimize Individual and Population Health." We are pleased to have the abstracts from these presentations compiled in one volume, and look forward to the 8th Annual Meeting, to be held in December in Washington, D.C.

